# Bilateral femoral neck stress fractures in elderly individuals: A case report and literature review

**DOI:** 10.1097/MD.0000000000034681

**Published:** 2023-09-15

**Authors:** Zhanglu Fang, Jianhua Cao, Xun Wang, Li Zhang

**Affiliations:** a Center for Plastic & Reconstructive Surgery, Department of Orthopedics, Zhejiang Provincial People’s Hospital, Hangzhou Medical College, Hangzhou, Zhejiang, China; b School of Medicine, Zhejiang Chinese Medical University, Hangzhou, China.

**Keywords:** femoral neck stress fracture, insufficiency fracture, osteoporosis

## Abstract

**Rationale:**

Bilateral femoral neck stress fractures are relatively rare injuries that occur frequently in military recruits, athletes and patients with osteoporosis, renal bone disease, metabolic bone disease, and chronic steroid use. Herein, a case of an elderly patient with bilateral femoral neck stress fractures is reported.

**Patient concerns:**

A 65-year-old man presented to the author’s hospital with right hip pain for over a month. The patient was a farmer, had a long history of field labor before the onset of pain, denied any history of trauma.

**Diagnosis:**

The patient was diagnosed with a right subcapital fracture of the femoral neck after examination. The patient complained of only right hip symptoms, and hip computed tomography showed no abnormalities in the left hip. A tension fracture of the left femoral neck was missed due to unawareness of the abnormal signal of the left femoral neck seen on right hip magnetic resonance imaging.

**Interventions:**

During the first hospitalization, the patient underwent total hip arthroplasty (THA) on the right hip. Two months after the operation, the patient started to have pain in the left hip and underwent left THA again for a displaced left femoral neck fracture.

**Outcomes:**

The patient eventually underwent bilateral THA surgery and had a satisfactory functional recovery. But the oversight in the diagnostic process led to the patient undergoing left THA that could have been avoided.

**Lessons:**

For patients who complain of hip pain but deny a history of trauma, we should be concerned about the presence of a hip fracture even if the patient’s radiograph does not report a positive result. The most sensitive method is bilateral magnetic resonance imaging examination of the hip. Femoral neck stress fractures require early diagnosis and treatment to prevent complications.

## 1. Introduction

According to the literature, stress fractures mostly occur in athletes, soldiers or elderly individuals. Femoral neck fractures represent 11% of all stress fractures in athletes.^[1]^ Fullerton and Snowdy classified stress fractures of the femoral neck as nondisplaced fractures on the compression side, nondisplaced fractures on the tension side or displaced fractures.^[2]^ Additionally, stress fractures can also be subdivided into fatigue fractures, where a normal bone is unable to respond adequately to abnormal stress (overload or overuse), and insufficiency fractures, where abnormal bone is unable to withstand normal stress.^[3]^ The most common symptom of a femoral neck stress fracture is pain in the hip and the inability to stand. Stress fractures in elderly patients often manifest as displaced fractures, which may be related to the decline in pain sensitivity in elderly individuals and inactive therapeutic interventions due to economic reasons.^[4]^ The treatment method for a femoral neck stress fracture needs to be considered according to the patient’s physical condition, economic condition, age, fracture type and other factors. The general treatment methods include conservative treatment, internal fixation with cannulated screws, and sliding hip screw fixation, but patients with hip joint deformities are generally recommended to undergo valgus osteotomy, hip replacement (including hemiarthroplasty and total hip arthroplasty [THA]) and so on.

We report the case of an elderly patient with bilateral femoral neck stress fractures. The first right THA was performed for the right subcapital fracture of the femoral neck. At that time, the nondisplaced left femoral neck fracture on the tension side was missed. Two months after the operation, the patient underwent left THA again for a displaced left femoral neck fracture.

## 2. Case presentation

A 65-year-old Chinese male presented at our hospital with a 1-month history of onset of right hip pain. The pain was tolerable at that time, and the pain worsened 2 weeks later. A pelvic X-ray showed a fracture of the right femoral neck. The patient was a farmer, and he denied a recent history of trauma, glucocorticoid intake or radiotherapy. He had been a smoker and heavy drinker for more than 20 years.

**Figure 1.**
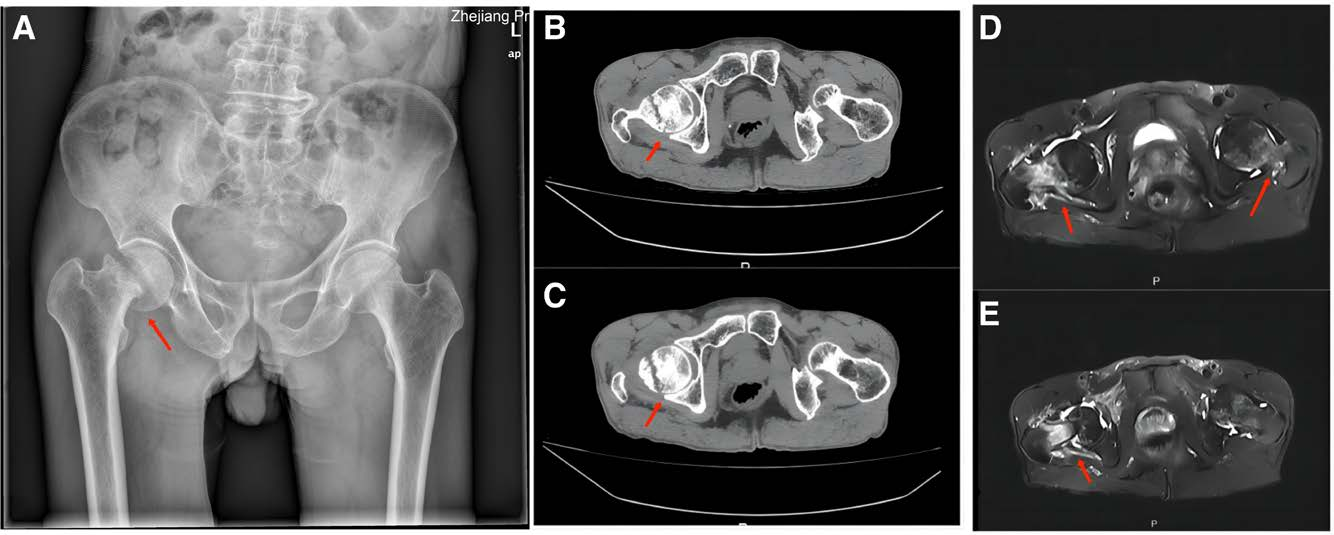
(A) Pelvic X-ray: An oblique translucent line was seen under the right femoral head, and the broken end was slightly separated and incarcerated. (B and C) Bilateral hip CT: the cortex of the right femoral neck was discontinuous, and the broken end was slightly angled and incarcerated. There was no obvious displaced fracture of the left femoral neck. (D and E) Right hip MRI: discontinuous cortical bone of the right femoral neck, edema of the surrounding bone marrow, displacement of the broken end, and swelling of the surrounding soft tissue. Left femoral neck edema with abnormal signal can be seen. CT = computed tomography.

The blood examination and biochemical laboratory values were within normal limits. Whole-body bone mineral densitometry (BMD) was measured by dual-energy X-ray absorptiometry to assess the mineralization status of bones. BMD measured at the L1–L4 level showed severe osteoporosis with a T score value of −4.1 and a Z score value of −2.6. At the left femoral neck level, the T score was −2.9 and the Z score was −1.7; at the right femoral neck level, the T score was −3.1 and the Z score was −1.9. The imaging examination is shown in Figure 1.

**Figure 2.**
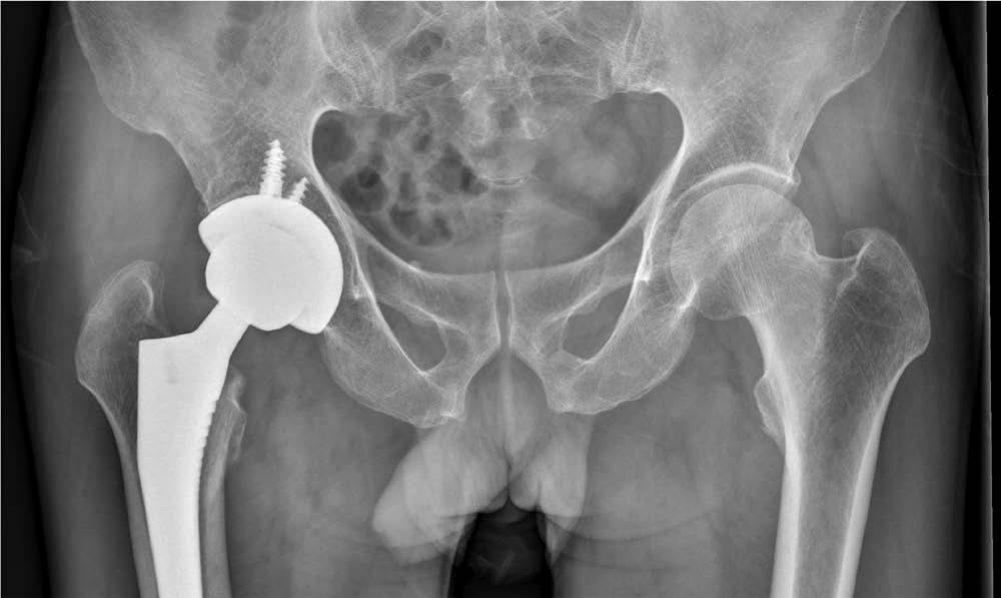
Postoperative pelvic X-ray showing good prosthesis position after right THA. No obvious displacement fracture of the left femoral neck was found.

We recommended that the patient undergo right-sided THA. The patient consented and underwent right THA through a posterolateral approach under general anesthesia. During surgery, we found sclerosis, steatosis and necrosis at the fracture end of the right femoral neck. Two days after surgery, the patient was asked to exercise his quadriceps and calf muscles in bed and use a walker to move with the assistance of a sports therapist. Meanwhile, postoperative pelvic radiographs showed that the THA surgery was very successful (Fig. 2). Overall, the patient recovered well during the hospital stay and was discharged home as normal. The first surgery went smoothly, and an intensive rehabilitation program began early. After his discharge from the hospital, oral calcium tablets and vitamin D were given for anti-osteoporosis treatment. Additionally, the patient was advised to undergo regular follow-up.

**Figure 3.**
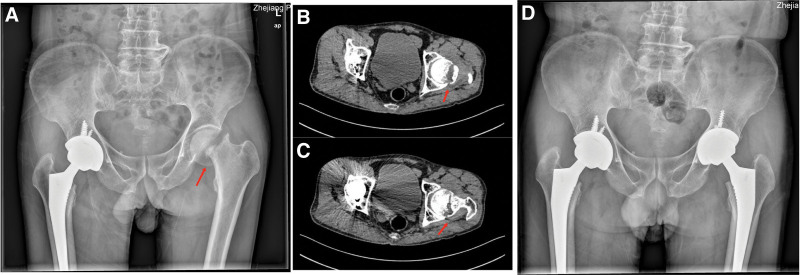
(A) Pelvic X-ray: left femoral neck fracture with dislocation and displacement. Good prosthesis position after right THA. (B and C) Bilateral hip CT: Displaced fracture of the left femoral neck with osteosclerosis of the broken end. (D) Postoperative pelvic X-ray: good prosthesis position after bilateral hip THA. CT = computed tomography, THA= total hip arthroplasty.

Two months after the operation, the patient was admitted to the hospital due to pain in the left hip. At the same time, he denied a history of trauma. The left lower limb could not be straightened when lying down. Blood examination and biochemical laboratory values were within normal limits. The imaging examinations are shown in Figure 3. After admission, the patient underwent left THA through a posterolateral approach under general anesthesia. Postoperative pelvic radiographs showed good positioning of the prosthesis after THA. Similarly, the patient underwent early muscle exercise and intensive rehabilitation training after surgery. After his discharge from the hospital, the patient received enhanced anti-osteoporosis treatment, including oral calcium tablets, vitamin D and a regular injection of denosumab.

## 3. Discussion

Femoral neck stress fractures are rare injuries. To the best of our knowledge, the literature reports related to femoral neck stress fractures are mostly case reports on soldiers and athletes, and there are few reports on femoral neck stress fractures in elderly patients.^[5–7]^ However, we found that these patients are the most accessible in the clinical setting. Therefore, it is crucial to diagnose these patients promptly and to intervene early and correctly. Failure to identify potential occult fractures in a timely manner may result in serious secondary injury to the patient.^[8]^ In this case, the patient had a left-sided tension femoral neck fracture that was not detected at the first visit, leading the patient to undergo THA surgery on the right side alone. Occult fractures that are not effectively treated slowly develop into displaced fractures through daily life.

Based on the literature summary, we reviewed the case reports of femoral neck stress fractures and the corresponding treatment plans (Table 1). The etiology of femoral neck stress fractures can be roughly divided into 2 categories. One type is a normal or abnormal stress repeatedly acting on a normal quality femoral neck, which can also be called a fatigue fracture and is usually seen in athletes and soldiers.^[3,5,28–32]^ Fatigue fractures may also be secondary to patients with abnormal femoral neck anatomy.^[11,33–35]^ The other type of fracture is an insufficiency fracture, in which the quality of the bone is reduced for various reasons, such as senile osteoporosis, postmenopausal osteoporosis, renal bone disease, vitamin D deficiency and other metabolic bone diseases, cortisol use, neurological sexual anorexia, and the use of certain drugs.^[9,21,25,26,36,37]^ Devas et al proposed the earliest classification method for femoral neck stress fractures, which are divided into 2 types according to whether the fracture end is displaced: Type I, simple compression fracture without displacement; and Type II, compression fracture with displaced fracture ends.^[38]^ Blickenstaff and Morris summarized a new classification method: Type I, no obvious fracture line on X-ray, only localized bone thickening in the femoral neck; Type II, fracture line visible on X-ray through the neck or spur of the femur without displacement of the fracture end; and Type III, completely displaced femoral neck fracture.^[39]^ Fullerton and Snowdy further proposed a new classification based on the fracture mechanism (compression and tension fractures) and fracture displacement: Type I, compression fracture of the medial femoral neck; Type II, tension fracture of the lateral femoral neck; and Type III, completely displaced femoral neck fracture.^[2]^ Early diagnosis of femoral neck stress fractures is actually difficult. There may be only partial osteosclerosis or no positive signs at all on pelvic X-ray. Therefore, further hip computed tomography and magnetic resonance imaging (MRI) scans are required when the patient indicates a history of hip pain without positive signs on the pelvic radiograph. Hip MRI is currently the most sensitive imaging test for diagnosing femoral neck stress fractures.^[40]^ For elderly patients, whole-body BMD is also necessary. We recommend further laboratory tests, such as serum vitamin D, bone metabolism indicators and serum electrolytes, for such patients. Treatment depends on the type of fracture. It is necessary to comprehensively consider the patient’s financial situation, age, physical condition and many other factors to formulate the most appropriate treatment plan for individual patients. In general, compression fractures can first be treated conservatively. However, conservative treatment of tension fractures has a high failure rate, and surgery is often considered after failure. Displaced fractures are recommended for surgical intervention. For younger patients, internal fixation with cannulated screws or sliding hip screw fixation is often the treatment of choice, while hip replacement is the usual option after failure of initial surgical treatment. Importantly, etiological treatment should be initiated promptly after surgical treatment to avoid subsequent fractures. For example, patients with osteoporosis require standardized anti-osteoporosis treatment. In addition, proper postoperative rehabilitation is essential. Some patients with a unilateral femoral neck stress fracture may develop a contralateral femoral neck stress fracture during postoperative rehabilitation due to stress changes.

**Table 1:** Case reports of bilateral femoral neck insufficiency fractures.

Author	Sex	Age (years)	Pathogenesis	Fracture classification	Treatment
Gupta^[9]^	Female	10	Vitamin D deficiency	Bilateral displaced fracture	L: valgus intertrochanteric osteotomy
					R: internal fixation with cannulated screw
Kalaci^[10]^	Female	18	Osteoporosis	Bilateral nondisplaced fracture (tension side)	Bilateral internal fixation with cannulated screw
Kerim^[11]^	Female	26	Vitamin D deficiency	Bilateral nondisplaced fracture (compression side)	L: DHS
					R: DHS
Santiago^[12]^	Female	27	Vitamin D deficiency	Bilateral nondisplaced fracture (compression side)	L: DHS
					R: DHS
Jacques^[13]^	Female	38	Osteoporosis and anorexia nervosa	Bilateral nondisplaced fracture (compression side)	L: DHS
					R: DHS
Tomofumi^[14]^	Female	41	Osteoporosis and vitamin D deficiency	Bilateral nondisplaced fracture	L: DHS
					R: DHS
Sivas^[15]^	Female	42	Osteomalacia	R: nondisplaced fracture (compression side)	Expectant treatment
				L: nondisplaced fracture	
Joseph^[16]^	Female	46	Osteoporosis and steroid use	Bilateral nondisplaced fracture (compression side)	L: DHS
					R: DHS
Haddad^[17]^	Female	56	Steroid use	L: displaced fracture	Bilateral internal fixation with cannulated screw
				R: nondisplaced fracture	
Tomar^[18]^	Female	58	Vitamin D deficiency	L: nondisplaced fracture (compression side)	L: THA
				R: nondisplaced fracture (tension side)	R: THA
Martin^[19]^	Female	65	Osteoporosis	R: displaced fracture	R: hemiarthroplasty
Chamseddine^[20]^	Female	71	Osteoporosis and vitamin D deficiency	L: displaced fracture	L: hemiarthroplasty
				R: nondisplaced fracture	R: Hemiarthroplasty
Sathyanarayana^[21]^	Male	23	Renal osteopathy	Bilateral displaced fracture	L: hemiarthroplasty
					R: hemiarthroplasty
Mariani^[22]^	Male	24	Vitamin D deficiency	Bilateral displaced fracture	L: THA
					R: THA
Chouhan^[23]^	Male	32	Osteoporosis and connective tissue tumor	Bilateral nondisplaced fracture (compression side)	Bilateral expectant treatment after tumor resection
Saisunder^[5]^	Male	35	ART treatment	L: displaced fracture	L: DHS
				R: nondisplaced fracture (compression side)	R: expectant treatment
Nagao^[24]^	Male	36	Osteoporosis and vitamin D deficiency	Bilateral displaced fracture	Bilateral hemiarthroplasty
Kiyokazu^[25]^	Male	51	Vitamin D deficiency and osteoporosis	Bilateral nondisplaced fracture (compression side)	Bilateral internal fixation with cannulated screw
Narra^[26]^	Male	52	Osteoporosis and HAART treatment	Bilateral displaced fracture	L: hemiarthroplasty
					R: expectant treatment
Tan^[27]^	Male	55	Osteoporosis	Bilateral displaced fracture	L: THA
					R: internal fixation with cannulated screw

DHS = dynamic hip screw, L = left side, R = right side, THA = total hip arthroplasty.

**Figure 4.**
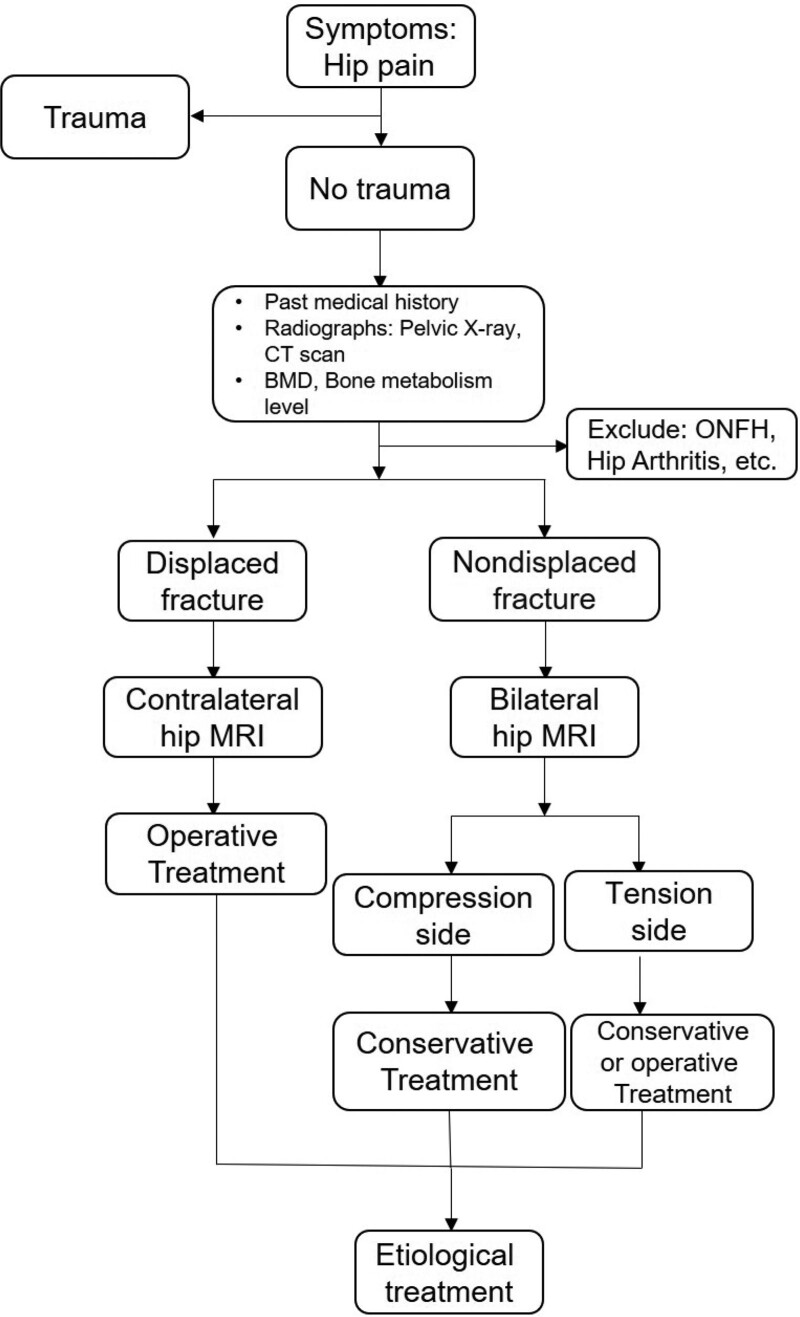
Recommended treatment options for patients with femoral neck stress fractures without a history of trauma. ONFH, osteonecrosis of the femoral head.

In our case, the patient had a 20-year history of smoking and drinking, which is one of the risk factors for osteoporosis.^[41]^ A complete BMD test at the time of admission can confirm the diagnosis of osteoporosis. The patient was a farmer, and his occupation required him to work in the fields for a long time. Therefore, we can speculate that the 2 together caused the bilateral femoral neck stress fracture in this patient, which can also be considered a type of insufficiency fracture. The patient should have been diagnosed with a left tension femoral neck fracture and a right displaced femoral neck fracture at the first visit. However, due to the lack of attention to the left femoral neck edema and abnormal signals indicated by the right hip MRI during the first treatment, no further MRI examination of the contralateral hip was performed, resulting in the missed diagnosis of left tension femoral neck fracture. Subsequently, during the rehabilitation period after the first THA, the patient was strengthened with muscle and pace exercises, which resulted in greater compression and load on the contralateral hip joint. The final result was that the left tension femoral neck fracture gradually developed into a displaced fracture. In this case, if abnormal edema signals in the left hip had been identified during the first hospitalization, early weight-bearing exercise would have been reduced after the right THA, and only muscle and nonweight-bearing training would have been performed. At the same time, intensifying the anti-osteoporotic therapy in this patient and regularly reviewing the progression of the left femoral neck nondisplaced fracture could have been one way to avoid a catastrophic outcome of the left femoral neck. Therefore, we recommend the following treatment options for patients with femoral neck stress fractures without a history of trauma (Fig. 4).

Overall, we can see from the treatment of this case that (1) in patients with hip fractures without a history of trauma, further investigations on bone metabolism, osteoporosis and hip MRI are required to avoid underdiagnosis of occult fractures. (2) Hip fractures among different ages, fracture types and etiologies require individualized treatment options. In elderly patients, we recommend hip replacement to avoid the possibility of failure after internal fixation and the need for reoperation. (3) It is necessary to carry out corresponding basic treatment and regular review according to the etiology of patients with femoral neck stress fractures after surgery.

## 4. Conclusion

For patients who complain of hip pain but deny a history of trauma, we should be concerned about the presence of a hip fracture even if the patient’s radiograph does not report a positive result. The most sensitive method is bilateral MRI examination of the hip. We recommend a protocol for the management of patients with stress fractures of the femoral neck without a history of trauma. Stress fractures of the femoral neck require early diagnosis and proper treatment, and the necessary etiological treatment is indispensable.

## Author contributions

**Conceptualization:** Li Zhang.

**Investigation:** Zhanglu Fang, Jianhua Cao.

**Visualization:** Xun Wang.

**Writing – original draft:** Zhanglu Fang, Xun Wang.

**Writing – review & editing:** Li Zhang.
